# Detrimental Effects of RNAi: A Cautionary Note on Its Use in *Drosophila* Ageing Studies

**DOI:** 10.1371/journal.pone.0045367

**Published:** 2012-09-17

**Authors:** Nazif Alic, Matthew P. Hoddinott, Andrea Foley, Cathy Slack, Matthew D. W. Piper, Linda Partridge

**Affiliations:** 1 Institute of Healthy Ageing, and GEE, University College London, London, United Kingdom; 2 Max-Planck Institute for the Biology of Ageing, ZMMK Forschungsgebäude, Köln, Germany; St. Georges University of London, United Kingdom

## Abstract

RNA interference (RNAi) provides an important tool for gene function discovery. It has been widely exploited in *Caenorhabditis elegans* ageing research because it does not appear to have any non-specific effects on ageing-related traits in that model organism. We show here that ubiquitous, adult-onset activation of the RNAi machinery, achieved by expressing a double stranded RNA targeting *GFP* or *lacZ* for degradation, or by increasing expression of Dicer substantially reduces lifespan in *Drosophila melanogaster*. Induction of *GFPRNAi* construct also alters the response of lifespan to nutrition, exacerbating the lifespan-shortening effects of food containing a high quantity of yeast. Our study indicates that activation of the RNAi machinery may have sequence-independent side-effects on lifespan, and that caution needs to be exercised when employing ubiquitous RNAi in *Drosophila* ageing studies. However, we also show that RNAi restricted to certain tissues may not be detrimental to lifespan.

## Introduction

Ageing and the associated functional decline are a major medical and socioeconomic concern for developed countries [1], [2]. Despite the complexity of the process, we now known that ageing can be modulated by pharmacological, genetic and environmental interventions [2]. This has resulted in an explosion in research activity aimed at identifying the genes, pathways and processes that can be used as potential targets for interventions into human ageing, to achieve better health for older people. These efforts have been successful in identifying numerous longevity determinants, especially in the simpler, genetically-amenable animal models such as the nematode worm *Caenorhabditis elegans* and the fruit fly *Drosophila melanogaster*.

One of the main tools used for knocking-down gene activity in *C. elegans* ageing research is RNA interference (RNAi). RNAi is an evolutionarily conserved phenomenon where the activity of a gene is silenced post-transcriptionally by means of mRNA degradation triggered by a short, double-stranded RNA (dsRNA) complementary in sequence to a part of the target message [3]. The capacity for RNAi is native to the cell and utilises its inherent processing and degradation machinery, including the endoribonuclease Dicer, that are shared with other pathways, such as the microRNA (miRNA) processing machinery [3], [4]. Importantly, RNAi can be triggered by exogenously-provided dsRNA and for this reason it has been widely exploited for gene-function analysis [3].

In worms, RNAi can be achieved by simply feeding them *Escherichia coli* expressing a suitable dsRNA [3]. RNAi has been so extensively used in *C. elegans* ageing research that even genome-wide RNAi screens for longevity have been performed, where RNAi knock-down of certain genes has been found to extend lifespan [5], [6]. Other studies have used RNAi to determine the genetic requirements for lifespan extension by environmental changes, such as dietary restriction [7].

In stark contrast, ubiquitous expression of an RNAi construct has rarely been reported as extending lifespan in *Drosophila*, with some notable exceptions (eg. see reference [8]), whereas loss of function mutants, or induction of dominant negative forms of proteins often do extend lifespan [9], [10], [11], [12]. The general paucity of reports of lifespan-extension by ubiquitous RNAi in *Drosophila* could be attributable to a correlated negative effect stemming from the activation of the RNAi machinery, coupled with a publication bias for negative results. Such a deleterious side-effect of RNAi could mask any beneficial effects resulting from the loss-of-function of the targeted gene. Indeed, here we report that ubiquitous expression of dsRNAs targeting a portion of the *GFP* or *lacZ* gene, or induction of Dicer, results in shortened lifespan in *Drosophila*. Furthermore, the expression of the same *GFP* RNAi construct changes the response of lifespan to diet. Our study reveals that induction of RNAi can have sequence-independent, detrimental effects in the fly, and highlights that caution needs to be exercised when using RNAi as a tool for longevity studies in *Drosophila*.

## Results

**Figure 1:**
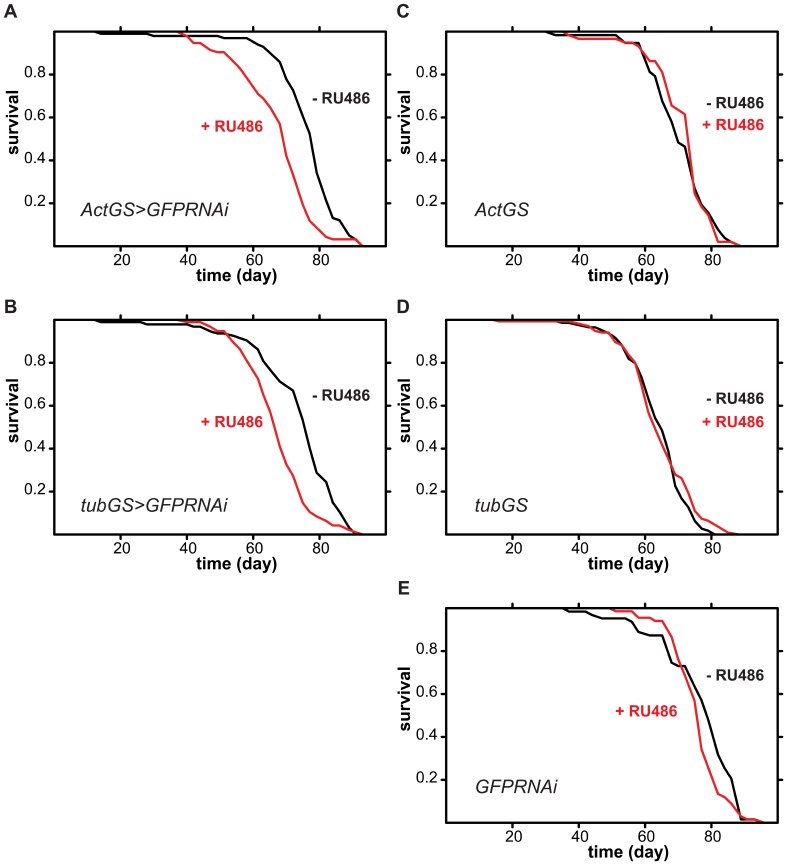
Ubiquitous adult-induced expression of dsRNA targeting *GFP* for RNAi shortens lifespan in *Drosophila* females. Mated female flies of the indicated genotype were fed 1 SYA food containing RU486 (red lines) or not (black lines) from day two of adulthood. **A** and **B** Log-rank test detected significant differences between the induced and uninduced conditions (p<0.0001, n =  ∼100 per condition). **C**, **D** and **E** Log-rank test did not detected significant differences between the induced and uninduced conditions (p>0.05, n =  ∼100 per condition).

To examine the effects of activation of the RNAi machinery on *Drosophila* lifespan, we chose a construct that produces a dsRNA with no target mRNA in the fly, an inverted repeat of 604 bp of the *GFP* gene sequence (*UAS-GFPRNAi*), which can efficiently knock-down GFP expression [13]. To avoid confounding developmental effects, we used GeneSwitch drivers that are only induced upon feeding the flies the RU486 steroid drug [14], [15]. We focused on the effects in female flies since they are most commonly used in ageing studies. Driving the expression of the construct with two frequently used ubiquitous GeneSwitch drivers, *actin* GeneSwitch (ActGS) and *tubulin* GeneSwitch (*tubGS*) from day two of adulthood resulted in substantial and significant reduction in lifespan (**Figure 1A and B**). Feeding RU486 to control flies containing only the driver or only the transgene had no effect on their lifespan (**Figure 1C, D and E**). Hence activation of the RNAi machinery appears to decrease lifespan in *Drosophila*.

**Figure 2:**
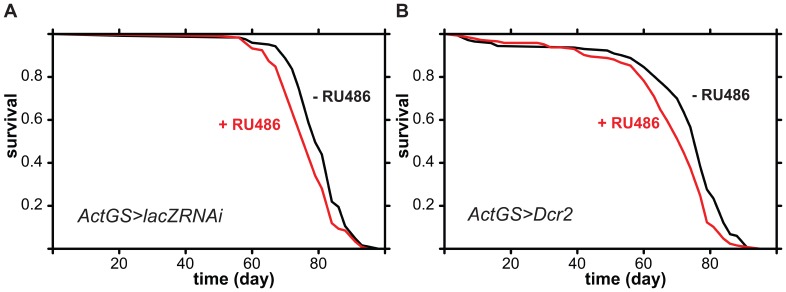
Ubiquitous adult-induced expression of dsRNA targeting *lacZ* for RNAi, or of *Dicer2*, shortens lifespan in *Drosophila* females. A Mated *ActGS>lacZRNAi* female flies of the indicated genotype were fed 1 SYA food containing RU486 (red lines) or not (black lines) from day two of adulthood. Log-rank test detected significant differences between the induced and uninduced conditions (p = 0.004, n =  ∼120 per condition). B Same as in A but for *ActGS>Dcr2*. Log-rank test detected significant differences between the induced and uninduced conditions (p = 0.0006, n =  ∼150 per condition).

We examined the sequence of the GFPRNAi construct with the dsCheck program [16] and found that it does not contain any 19-mers that perfectly match to a part of the *Drosphila* genome. This indicated that the observed detrimental effect on lifespan was sequence-independent. To confirm this, we ubiquitously expressed an RNAi construct targeting the bacterial *lacZ* transcript (*UAS-lacZRNAi*) with the ActGS driver (**Figure 2A**), as well as activated the RNAi machinery not by inducing an exogenous dsRNA but by induction of Dicer, an endoribonuclease encoded by the *Dcr2* gene (**Figure** **2B**). Both these manipulations shortened lifespan, confirming that the reduction in lifespan results from sequence-independent effects of the activation of the RNAi machinery.

**Figure 3:**
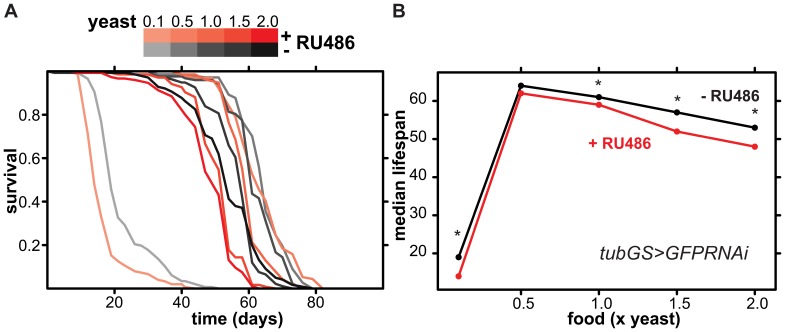
Lifespan-shortening effects of ubiquitous adult-induced expression of *UAS-GFPRNAi* are diet-dependent. Mated female *tubGS>GFPRNAi* flies were reared on 1 SYA food and placed on different foods containing increasing amounts of yeast (0.1× to 2× relative to standard food) either containing RU486 (shades of red) or not (shades of grey) from day two of adulthood. **A** The resulting lifespans. **B** The median survival times, with asterisks indicating significant differences in survival between the induced (+ RU486) and uninduced (-RU486) conditions (Log-rank test, p<0.001, n =  ∼150 per condition). The survival between induced and uninduced at 0.5× yeast was not significant (Log-rank test, p>0.5, n =  ∼150 per condition). Cox Proportional Hazards analysis revealed significant effect of yeast quantity, presence of RU486 and their interaction (p<0.0001).

RNAi has also been used in the worm to test if particular genes are involved in mediating the effects of dietary restriction, a phenomenon where lifespan is maximised at intermediate food concentrations [7]. In *Drosophila*, dietary restriction can be achieved by dilution of the yeast component of fly food [17]. To assess if RNAi affected the response of the flies to dietary restriction, we investigated whether the flies subjected to RNAi differed from controls in their lifespan response. Ubiquitous expression of the *GFPRNAi* construct with the *tubGS* driver exacerbated the detrimental effect of RNAi on lifespan at higher and lower levels of yeast in the food, so that the two acted synergistically to shorten lifespan (**Figure 3A and B**). RNAi did not significantly shorten lifespan at its peak (0.5× yeast) but was detrimental at higher and lower yeast concentrations (**Figure 3B**), including the 1× yeast concentration that was tested above (**Figure 1**). Analysing the data with Cox Proportional Hazard analysis [18] confirmed this, and both the effect of food or transgene induction were significant and so was their interaction (p<0.0001). Hence, activation of the RNAi machinery appears to alter the lifespan response to different yeast concentrations in *Drosophila*.

**Figure 4:**
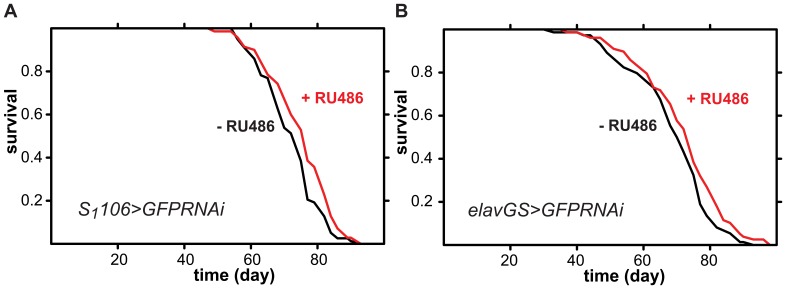
Adult-induced expression of dsRNA targeting *GFP* for RNAi in midgut and abdominal fat body or in neurons does not shorten lifespan in *Drosophila* females. Mated female flies of indicated genotype were fed 1 SYA food containing RU486 (red lines) or not (black lines) from day two of adulthood. A Log-rank test detected a slight but significant increase in lifespan in the induced compared to the uninduced condition (p = 0.04, n =  ∼80 per condition). B Log-rank test did not detected significant differences between the induced and uninduced conditions (p>0.05, n =  ∼80 per condition).

In *Drosophila* RNAi is potentially useful for tissue-restricted loss-of-function studies, so we wanted to examine if this, also, is necessarily detrimental to *Drosophila* lifespan. To assess this, we tested two GeneSwitch drivers with tissue-restricted expression that are commonly used in ageing studies: *S_1_106*, which drives expression in the midgut and abdominal fat body, and *elavGS*, which drives expression in neuronal cells [19]. Driving the expression of the *GFPRNAi* construct with either driver had no negative effect on lifespan (**Figure 4A and B**). Hence, localised activation of the RNAi machinery does not necessarily have a detrimental effect on lifespan. However, driving *GFPRNAi* with *S_1_106* showed a slight but significant increase in survival (**Figure 4A**), indicating that caution will need to be exercised when employing tissue-specific drivers as well, on a case-by-case basis.

## Discussion

We show here that ubiquitously driving a construct expressing dsRNA containing a portion of the GFP or LacZ protein-coding sequence, or ubiquitous induction of Dicer, has a negative effect on *Drosophila* lifespan. Several unwanted effects of exogenously induced RNAi are known. Sequence-specific off-target events can be observed in both mammals and flies where, for example, the presence of short sequences homologous to a gene non-intentionally targeted by the construct can result in its degradation [20]. In *Drosophila*, the occurrence of these sequence-dependent off target effects correlates with the presence of 19mers in the dsRNA that are complementary to a non-intentionally targeted mRNA, resulting in its degradation [21]. Since the GFPRNAi construct contains no such 19mers and the negative effect on lifespan can be recapitulated using RNAi against *lacZ* or by Dicer over-expression, this reduction in lifespan is unlikely to result from sequence-dependent, off-target effects; rather it reveals that activation of the RNAi machinery may have sequence-independent, negative effects on *Drosophila* lifespan. At least two such sequence-independent side effects are documented in mammals. Firstly, dsRNA of certain lengths can result in activation of the interferon or Tol-like receptor response [20]. Secondly, high levels of expression of short hairpin RNAs used for RNAi can result in depletion of the components of the processing machinery, severely compromising cellular functions and causing organ damage *in vivo* [22]. In particular, high levels of expression of short hairpin RNAs in mouse liver have been shown to result in damage to liver tissue and ultimately to death of the mouse. This effect appears to be mediated by depletion of the dsRNA processing machinery away from its normal cellular roles such as processing of miRNAs [22]. A recent report has revealed the important role of miRNAs in *Drosophila* ageing [23] and so a similar phenomenon may underlie the lifespan shortening effect of RNAi in *Drosophila*.

Importantly, our study reveals that caution needs to exercised when using RNAi for lifespan assays in *Drosophila* and that, in addition to the sequence-specific off-target effects, sequence-independent effects also have to be controlled for. In particular, future studies will require employment of a non-specific RNAi construct, such as *UAS-GFPRNAi*, as a control treatment.

## Materials And Methods

The *UAS-GFPRNAi* construct [13] and *UAS-Dcr2* were obtained from the Bloomington stock center, *tubGS* (reported in [24]) was a kind gift of Scott Pletcher and ActGS [25] was a gift of John Tower. *S_1_106* and *elavGS* have been described [19], [26]. All of the above constructs were backcrossed at least 6 times into a Dahomey background carrying a *w^1118^* mutation and that had been cured of *Wolbachia* infection and frequently out-crossed to the Dahomey population housed in a population cage. *UAS-lacZRNAi* construct [27] was a kind gift of Martin Junger and Hugo Stocker. This construct was not backcrossed but the *ActGS>lacZRNAi* flies were created in a hybrid background using the backcrossed *ActGS* virgins and the lifespan of resulting progeny determined on food containing or not RU486. Stocks were maintained and experiments conducted at 25°C on a 12 h:12 h light:dark cycle at constant humidity using standard sugar/yeast medium (1 SYA) [28]. The food contained 5% sucrose (w/v), 1.5% agar (w/v), 0.3% propionic acid (v/v), 0.3% nipagen (w/v) and either 1% (0.1× yeast), 5% (0.5× yeast), 10% (1× yeast) or 20% (2× yeast) autolysed brewer's yeast (w/v) and was prepared as previously described [26]. The food with 1× yeast is referred to as 1 SYA. For lifespan assays, females were separated from males on day 2 of adulthood, transferred to food containing zero or 200 µM RU486, housed 10 per vial and transferred to new food 3 times per week. Statistical analysis was performed in JMP (version 9) software (SAS Institute) or Excel (Microsoft).
